# Exploring the mental health impact on private flat owners in residential buildings with external combustible cladding

**DOI:** 10.1192/bjo.2021.715

**Published:** 2021-06-18

**Authors:** William Martin

**Affiliations:** Warrington & Halton NHS Trust, UK Cladding Action Group

## Abstract

**Aims:**

To explore the mental health of private flat owners in residential buildings with external combustible cladding, which require remediation following the 2017 Grenfell tower fire.

The speed at which the fire at Grenfell spread, killing 72 people, is largely attributed to external combustible cladding. It is increasingly suspected that there are ongoing profound effects on the mental health of people living in flats with external combustible cladding like Grenfell both in the UK and abroad. The issue has left flat owners facing severe financial hardship, the threat of bankruptcy and concerns about safety in their own homes.

**Method:**

An exploratory ‘Google Forms’ online mental health survey comprising multiple choice and free text questions over 47 sections was distributed to flat owners in affected buildings. The survey remained open for 6 weeks to allow response. 550 individual responses were studied.

**Result:**

550 individuals completed the survey, from 143 buildings across 45 UK councils.

As a direct result of external combustible cladding:

- 89.5% said their mental health had deteriorated,
- 22.5% reported having suicidal feelings or a desire to self-harm,
- 71.1% reported having difficulty sleeping,
- 93.8% said they were suffering from worry and anxiety,
- 59.6% used coping strategies to deal with their situation,
- 35.1% said that existing physical and mental health conditions had been exacerbated,
- 84.1% said they cannot move on with their lives and
- 57.9% of people had concerns about seeking help or treatment for mental/physical health problems caused by their situation during the pandemic.

In addition, free text responses reflected feelings of anxiety and low mood attributed to the constant fear of fire, and an inability to plan families and future homes. One person said, “I have been left utterly broken by this. My mental and physical health has worsened, I have severe anxiety, depression and PTSD. I struggle each day to keep myself alive.”

**Conclusion:**

Safe housing is a basic human right. The results show the current situation is having a detrimental impact on flat owners’ mental health and makes a strong case for the provision of specific services offering support - particularly given it is 3.5 years since Grenfell and a viable solution for all is yet to be found.

